# Psychiatric disorders in the acoustic neuroma: about a case

**DOI:** 10.11604/pamj.2019.33.80.18398

**Published:** 2019-06-04

**Authors:** Samira Younes, Sabria Khouadja, Samia Younes, Rim Ben Soussia, Walid Bouali, Ahmed Haj Mohamed, Lazhar Zarrouk

**Affiliations:** 1 Department of Psychiatry, University Hospital of Tahar Sfar, Mahdia, Tunisia; 2 Department of Neurology, University Hospital of Tahar Sfar, Mahdia, Tunisia

**Keywords:** Neurofibromatosis type 2, vestibular schwannoma, acoustic neuroma, hearing loss, psychiatry, psychosis

## Abstract

Neurofibromatosis type 2 (NF2) is a rare autosomal dominant disorder characterized by formation of central nervous system tumors. They are associated to significant morbidity due to multiple problems such as hearing loss that can lead to many psychiatric disorders.

## Introduction

Within the clinical picture of neurofibromatosis type 2 (NF2), vestibular schwannomas (or acoustic neuromas) are usually those with the greatest repercussion on a patient's quality of life. Given that patients can show non-specific symptoms for a long time, the diagnosis of the disease can be delayed for several years.

## Patient and observation

**Figure 1:**
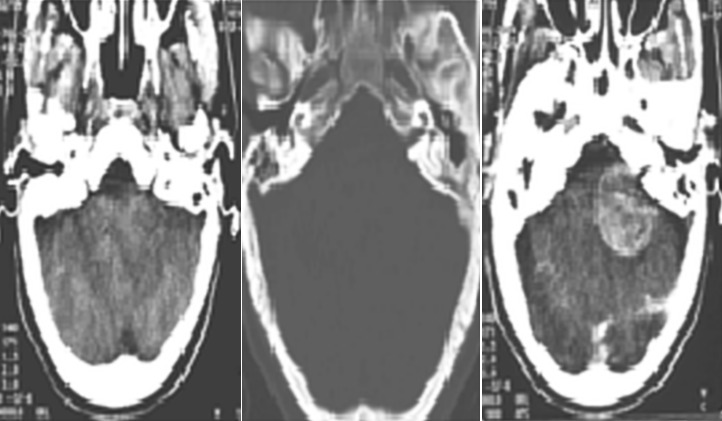
Axial section of the brain scan showing tumor of the left cerebellopontine angle extending to the left auditory meatus

This is the case of a 21-year-old patient who was admitted to our ward under the state hospitalization mode for instability, emotional and behavioral disorder. Our patient had a medical history of a one-sided deafness treated with a hearing prosthesis. For his psychiatric history, he was followed irregularly by a free-lance psychiatrist. The beginning of trouble dated back to 3 years marked by the installation of behavioral disorders such as fugue, agitation, irritability, sleep disorder and physical carelessness. A worsening of symptoms is 3 months, stated by the appearance of hostility and delusion of persecution towards his mother. The patient declines to eat the food that his mother cooked for him. He threatened her with a knife and he asked his sister to rewash his clothes. Overall, the clinical overview includes a delirium, clastic agitation strikes, emotional lability, cerebral ataxia and conjunctival hyperemia. A brain scanner was requested because of clinical presentation and the normality of laboratory tests, but also psychotropic intolerance including antipsychotics. The brain scan showed an association of bilateral acoustic neuromas, cavernous and intraventricular meningioma (Figure 1). These clinical and radiological signs met the diagnosis criteria for NF2 according to the consensus conference of the National Institute of Health in Bethesda established in the USA in 1988.

## Discussion

NF2, secondary to a defection in chromosome 22, is characterized by the appearance of schwannomas of the 8^th^ cranial nerve, as well as ocular alterations and meningiomas. NF2 is a rare clinical entity. According to recent studies, incidence of 1:25 000 and prevalence greater than 1:80 000 are estimated [1, 2]. The onset of NF2 is usually around 18-24 years, although the range is from birth to 70 years [3]. The most common symptoms are the appearance of neurological, ocular and cutaneous. Among the neurological lesions, the most observed characteristics are bilateral vestibular schwannomas. Initial symptoms include tinnitus, hearing loss and alterations in balance that begin insidiously; however, hearing loss may occur suddenly [4]. Treatment for the various symptoms of NF2 is similar for sporadic tumors of the same type. However, multidisciplinary management is obligatory to minimize disabilities and to avoid morbidity secondary to treatment of these tumors. The main prognostic factors are mean age at diagnosis, presence of intracranial meningiomas and treatment in specialized centers [5].

Hearing aids may be helpful early in the course of the disease [6]. The treatment of hearing loss should include patient referral to an audiologist and for lip-reading training, as well as hearing aids like cochlear implants or brainstem implants [6]. Kraepelin [7] was the first to describe the presence of paranoia and persecutory delusions in patients with impaired hearing. In a more recent meta-analysis of epidemiologic studies, the investigators found an increased risk of hearing impairment for all psychosis outcomes, such as hallucinations, delusions, psychotic symptoms and delirium, although the odds ratios were relatively small. Early exposure to hearing impairment led to elevate the risk of later development of schizophrenia. The investigators suggested that potential mechanisms underlying this association include loneliness and disturbances of source monitoring (locating the source of sounds and interpreting these sounds) [8]. In another study, self-reported hearing impairment was associated with increased frequency of psychotic symptoms among those using a hearing aid in younger persons but not older adults [9].

Depression is a particularly important construct to examine given anecdotal evidence from a number of treatment centers of increased rates of suicide among acoustic neuroma patients compared to the general population [10]. Furthermore, in a recent focus group study among acoustic neuroma patients, an association was found between severe postoperative headaches, depression and suicidal ideation [11]. Thomas and colleagues [12] found a fourfold increase in scores above a clinically significant cutoff for anxiety and depression symptoms among patients with a hearing impairment compared with the general population. In another study among community respondents in underserved areas and using self-rated scales, subjects who reported sensory loss had high rates of depression and a compromised quality of life compared with respondents without these impairments [13]. In yet another study, investigators explored the associated relationship of hearing impairment with anxiety symptoms. They found that, compared with individuals with no hearing impairment, the odds of prevalent anxiety were significantly higher among individuals with mild hearing impairment, and for those with moderate or severe impairment the odds were even greater. Hearing aid use was not significantly associated with lower likelihood of anxiety [14].

## Conclusion

Early diagnosis, multidisciplinary management in specialized units and improvements in treatment could increase hope and quality of life for the patients.

## Competing interests

The authors declare no competing interests.

## Authors’ contributions

All authors have contributed to this article and have read and agreed to the final manuscript.
